# Taming Macrophage Inflammasomes at the Sepsis—Lung Injury Interface: Lessons from Bats

**DOI:** 10.3390/biomedicines14102325

**Published:** 2026-10-13

**Authors:** Shunyao Chen, Jialiu Luo, Peidong Zhang, Shuaipeng Gu, Youxie Shen, Zhiqiang Lin, Yuhao Wu, Cong Zhang, Zhaohui Tang

**Affiliations:** 1 Department of Trauma Surgery, Emergency Surgery & Surgical Critical, Tongji Trauma Center, Tongji Hospital, Tongji Medical College, Huazhong University of Science and Technology, Wuhan 430030, China; tjchensy@hust.edu.cn (S.C.); luojialiu@hust.edu.cn (J.L.); peidongzhang@hust.edu.cn (P.Z.); gusp@hust.edu.cn (S.G.); shenyouxie@hust.edu.cn (Y.S.); linzhiqiang@hust.edu.cn (Z.L.); wuyuhao@hust.edu.cn (Y.W.); 2 Department of Emergency and Critical Care Medicine, Tongji Hospital, Tongji Medical College, Huazhong University of Science and Technology, Wuhan 430030, China

**Keywords:** septic lung injury, macrophage inflammasome, NLRP3–ASC axis, bat immunity, disease tolerance

## Abstract

Sepsis-associated lung injury remains a major cause of respiratory failure and death, yet treatment is still largely supportive once inflammatory tissue damage is established. Macrophage inflammasomes, especially the NLRP3–ASC–caspase-1 axis, occupy a central but paradoxical position in this process. They contribute to pathogen sensing and antimicrobial defense, but excessive or poorly timed activation promotes IL-1β and IL-18 release, gasdermin D-mediated pyroptosis, extracellular ASC speck propagation, neutrophil amplification, barrier disruption, alveolar edema and acute respiratory distress syndrome. Therefore, a clinically meaningful strategy may not be to abolish inflammasome activity, but to tame it. Bats provide a natural model of this principle. Despite hosting many viruses and maintaining high metabolic activity, bats limit inflammatory execution through dampened NLRP3 responsiveness, altered DNA inflammasome sensing, restrained caspase-1/IL-1β processing and ASC2-mediated inhibition of ASC-dependent inflammasomes, while preserving antiviral readiness. These findings suggest a host-defense-preserving framework for septic lung injury. This review proposes bat-inspired design rules: raising inflammasome activation thresholds, braking ASC-dependent propagation, limiting lytic effector output, targeting inflammasome-active macrophage states instead of macrophages as a bulk population, and applying biomarker-guided timing to avoid intervention during immune paralysis. We also discuss the need to define an “inflammasome-high septic lung injury” phenotype using lung injury progression, inflammasome output markers, macrophage-state signatures and immune competence indicators. Bats do not provide a ready-made therapy, but they offer a useful biological lesson: damaging inflammatory amplification can be restrained without erasing host defense.

## 1. Introduction: The Problem of Untamed Macrophage Inflammation in Septic Lungs

Sepsis is life-threatening organ dysfunction caused by a dysregulated host response to infection [1]. The lung is an early target: sepsis-associated lung injury and acute respiratory distress syndrome (ARDS) feature inflammation, alveolar-capillary leak and impaired gas exchange [2,3]. Treatment remains largely supportive once inflammatory lung damage is established [4].

Macrophages sit close to the center of this problem. In the healthy lung, alveolar macrophages quietly survey the airspace, remove debris, and maintain immune restraint. In septic lungs, they sense microbial and damage signals, recruit leukocytes and shape inflammation [5]. This response is needed. Without it, microbial control would fail. Yet when macrophage activation is excessive or poorly timed, the same system that protects the host begins to injure the lung.

Inflammasomes provide a useful lens through which to understand this switch. The NOD-like receptor family pyrin domain containing 3 (NLRP3) inflammasome, in particular, integrates microbial signals, extracellular adenosine triphosphate (ATP), ion fluxes, mitochondrial damage, and oxidative stress. Once activated, NLRP3 promotes apoptosis-associated speck-like protein containing a CARD (ASC) speck formation, caspase-1 activation, maturation of Interleukin (IL)-1β and IL-18, and gasdermin D-mediated pyroptosis [6,7]. These events can help alert the immune system to danger, but they also amplify inflammation in a tissue that has little tolerance for swelling, leakage, and cellular debris. In septic lungs, macrophage inflammasomes may therefore act less like a simple alarm system and more like a faulty amplifier—useful at first, harmful when left unchecked [8].

This duality partly explains why broad anti-inflammatory approaches in sepsis have been disappointing. Inflammation is not a uniform enemy. It changes over time, differs between patients, and may coexist with immune paralysis in the same clinical syndrome [9]. Complete suppression of inflammasome signaling could, in theory, reduce lung injury; it could also weaken host defense. A more attractive goal is not to abolish macrophage inflammasomes, but to tame them: to preserve their protective sensing functions while restraining the self-propagating loops that drive immunopathology.

Bats offer an unexpectedly elegant model for this idea. They can asymptomatically host many viruses that cause severe disease in humans, and they also display unusual longevity despite high metabolic demands. A key part of this biology appears to be controlled inflammation rather than indiscriminate immune activation [10]. Ahn and colleagues showed that bat immune cells have dampened NLRP3 inflammasome activation, with reduced ASC speck formation, lactate dehydrogenase (LDH) release, and IL-1β secretion after sterile and viral stimuli, without a major increase in viral load [11]. More recently, the same research program identified bat ASC2 as a potent endogenous suppressor of ASC-dependent inflammasomes, capable of reducing sterile and virus-induced inflammation in experimental models [12]. These findings do more than explain bat biology. They raise a translational question: can lessons from bats help us restrain macrophage inflammasomes in septic lungs without dismantling host defense?

This review follows that question. Rather than cataloguing all available inflammasome inhibitors, this review proposes a bat-inspired inflammasome-taming framework for septic lung injury—one that aims to preserve macrophage-mediated host defense while restraining the self-amplifying inflammatory circuits that damage the lung.

## 2. Macrophage Inflammasomes in Sepsis-Associated Lung Injury

Macrophage inflammasomes have measurable pulmonary consequences. In ARDS, Caspase-1 (CASP1), IL1B and IL18 transcripts and plasma IL-18 are increased and associate with adverse outcomes; in experimental lung injury, IL-18 or caspase-1 deficiency reduces inflammatory-cell influx, protein leakage and edema [13]. This is not just pathway speculation. It is lung pathology.

**Figure 1:**
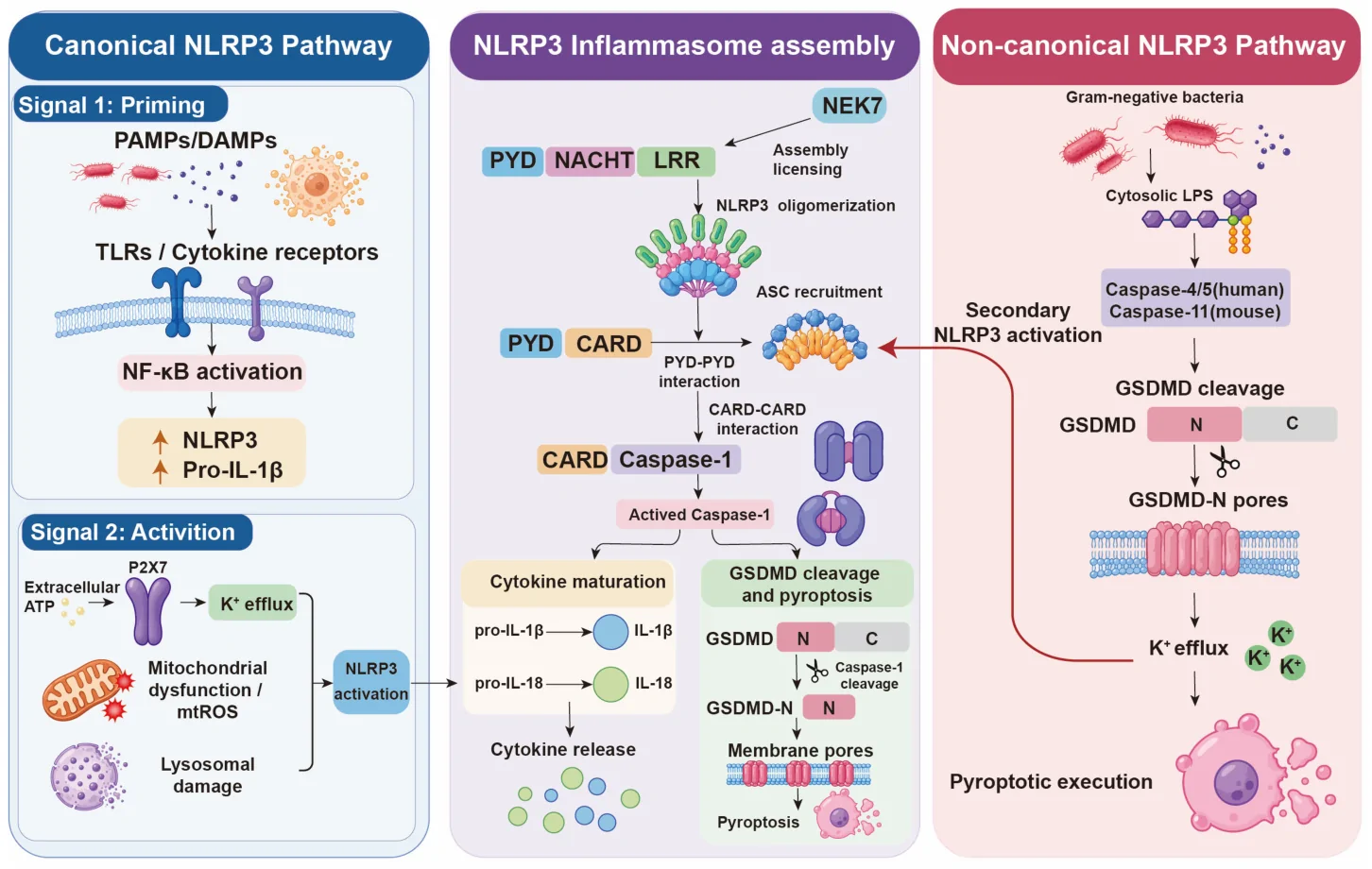
Canonical and non-canonical NLRP3 inflammasome activation in bacterial sepsis.

Mechanistically, the NLRP3–ASC–caspase-1 axis couples priming to cytokine maturation and Gasdermin D (GSDMD)-dependent pyroptosis [6,7]. Canonical NLRP3 assembly and its convergence with non-canonical cytosolic lipopolysaccharide (LPS) sensing are summarized in Figure 1. Genome-wide screening helped define the execution step: Gsdmd-deficient macrophages resisted pyroptosis induced by cytosolic LPS and by NLRP3, Absent in Melanoma 2 (AIM2), NLR family CARD domain-containing protein 4 (NLRC4), and Pyrin inflammasome stimuli, with markedly reduced IL-1β release despite preserved upstream signaling [14,15].

ASC specks make this response linger. Franklin et al. showed that extracellular ASC specks accumulate after inflammasome activation, correlate with LDH release, retain the capacity to process pro-IL-1β/pro-caspase-1, and can be engulfed by macrophages to trigger lysosomal damage and secondary IL-1β activation [16]. In a leukocyte-rich septic lung, that is a dangerous form of cell-to-cell communication.

Canonical NLRP3 activation involves nuclear factor (NF)-κB-dependent priming followed by NLRP3–ASC–caspase-1 assembly, leading to IL-1β/IL-18 maturation and GSDMD-mediated pyroptosis. Cytosolic LPS activates caspase-4/5 in humans or caspase-11 in mice, promoting GSDMD pore formation, K^+^ efflux, and secondary NLRP3 activation.

**Figure 2:**
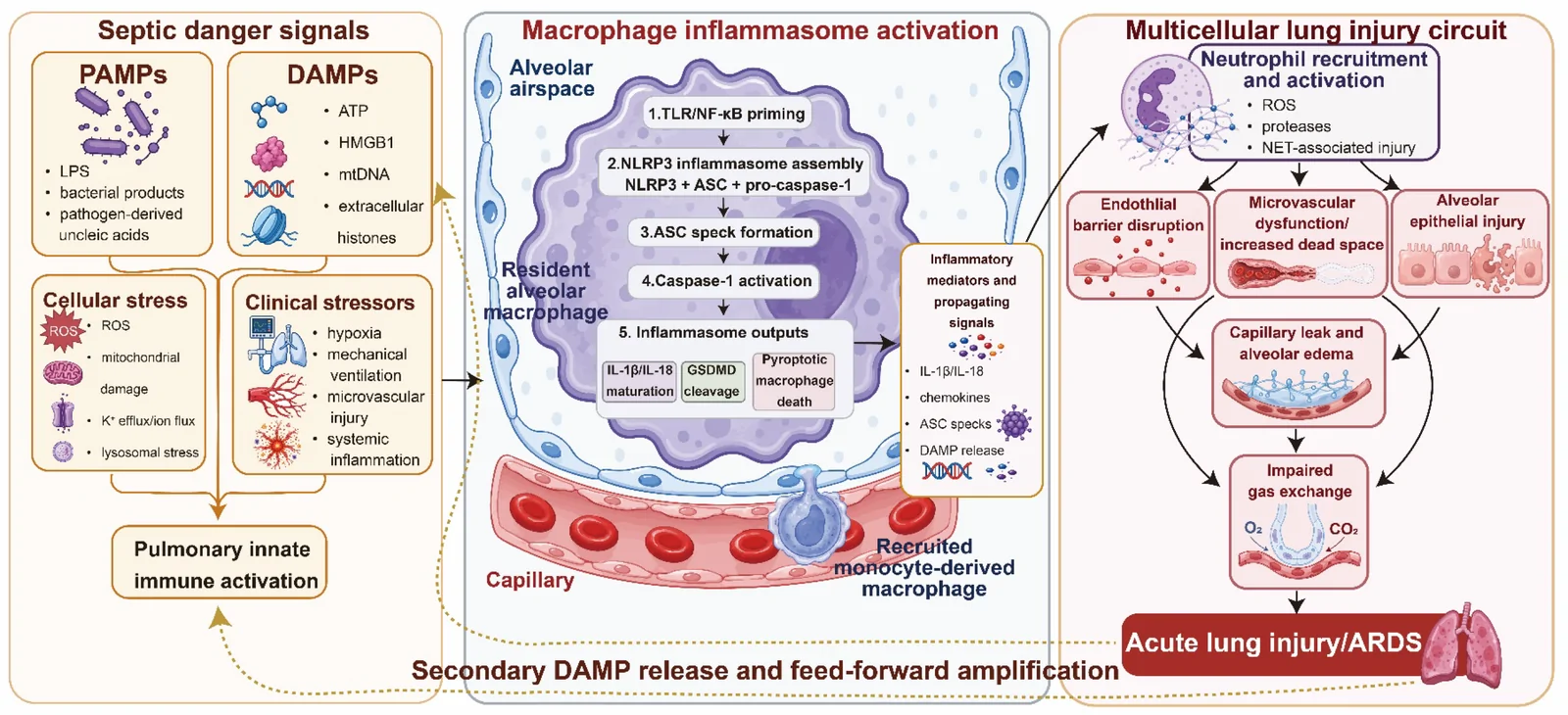
Macrophage inflammasome-driven multicellular injury circuit in septic lungs.

Single-cell studies now place these signals into discrete myeloid states. Wang et al. profiled 25,044 lung cells in cecal ligation and puncture (CLP)-induced lung injury and identified monocyte/macrophage subsets, including alveolar macrophages, MacroCxcl3, and Mono1; notably, Mono1 was most closely associated with the septic lung injury phenotype and expressed inflammatory mediators such as S100a8/S100a9, Hmgb1, and Mmp8 [17]. Another macrophage-focused scRNA-seq analysis resolved pulmonary macrophages into MAC1–MAC8 and found that MAC1 and MAC2 increased during sepsis and sepsis-48 h, with higher expression trends of *Nlrp3*, *Casp1*, *Gsdmd*, *Il1b*, and oxidative-stress-related genes *Ncf1* and *Txnip* [18]. Zhao et al. similarly showed that cleaved GSDMD and terminal deoxynucleotidyl transferase dUTP nick end labeling (TUNEL) signals were enriched mainly in F4/80+ pulmonary macrophages, with lung NLRP3, caspase-1, GSDMD, and cleaved GSDMD peaking around 24 h after CLP [19]. Downstream, neutrophils translate this macrophage alarm into physiology: intravital imaging revealed LPS-induced pulmonary capillary dead spaces, reduced functional capillary ratio, hypoxemia, and Mac-1-dependent neutrophil sequestration [20]. Macrophage inflammasomes therefore participate in a multicellular injury circuit involving recruited neutrophils, the pulmonary microvasculature, and other inflammatory compartments (Figure 2).

This pulmonary circuit should, in turn, be considered within a broader systemic myeloid–vascular network. Sepsis activates tissue-resident macrophage populations beyond the lung, including hepatic Kupffer cells, which participate in pathogen clearance, endotoxin handling, and systemic inflammatory regulation. Recent experimental evidence further indicates that sepsis can induce NLRP3- and GSDMD-associated pyroptotic loss of Kupffer cells, linking extrapulmonary macrophage inflammasome activity to systemic immune dysfunction [21]. In parallel, pulmonary microvascular endothelial cells are both targets and amplifiers of inflammatory injury. Experimental LPS-induced acute lung injury has linked TXNIP/NLRP3 inflammasome activation in pulmonary microvascular endothelial cells to increased endothelial permeability and lung injury [22]. NLRP3-associated endothelial pyroptosis has also been implicated in experimental sepsis-induced acute lung injury, where impaired mitochondrial quality control promoted endothelial inflammasome activation and permeability defects [23]. These observations indicate that pulmonary macrophage inflammasome activity occurs within a broader myeloid–endothelial inflammatory network rather than as an isolated alveolar process.

Inflammasome activation in sepsis is also shaped by cellular stress and metabolic reprogramming rather than by Toll-like receptor (TLR)–NF-κB signaling alone. Hypoxia and inflammatory metabolic remodeling can stabilize hypoxia-inducible factor 1-alpha (HIF-1α) and interact with NF-κB-dependent transcriptional programs, promoting glycolytic and pro-inflammatory macrophage states and increasing the capacity for IL-1β production [24]. Mitochondrial dysfunction, reactive oxygen species (ROS) accumulation, ionic disturbance, and release of mitochondrial components provide additional signals that can facilitate NLRP3 activation [7]. Importantly, these pathways participate in a feed-forward rather than strictly linear process. Once inflammatory caspases activate GSDMD, membrane pore formation and pyroptotic cell lysis can release inflammatory mediators and intracellular Damage-associated molecular patterns (DAMPs), including High mobility group box 1 (HMGB1) and other cellular contents [14,25]. These signals can further stimulate neighboring immune and endothelial cells, thereby amplifying pulmonary inflammation.

Inflammasome activation in septic lung injury should therefore not be interpreted exclusively as either a cause or a consequence of tissue damage. Rather, it can occupy both positions within a feed-forward injury circuit. Infection-associated Pathogen-associated molecular patterns (PAMPs) and early cellular stress can initiate inflammasome activation, whereas subsequent epithelial and endothelial injury, mitochondrial dysfunction, extracellular ATP release, and other DAMPs can further amplify NLRP3 signaling. In turn, caspase-1 activation, IL-1β/IL-18 maturation, GSDMD-mediated pyroptosis, and secondary DAMP release can propagate leukocyte recruitment, barrier dysfunction, and tissue injury. Thus, the relative contribution of inflammasome signaling as an initiating driver versus a downstream amplifier is likely to vary with the infection model, cellular compartment, and disease stage.

In sepsis, pulmonary macrophages integrate diverse danger cues, including pathogen-associated molecular patterns (PAMPs), damage-associated molecular patterns (DAMPs), oxidative and mitochondrial stress, and clinical stressors such as hypoxia and mechanical ventilation. These inputs promote TLR/NF-κB priming and activation of the NLRP3 inflammasome, leading to ASC speck formation, caspase-1 activation, maturation of IL-1β and IL-18, GSDMD cleavage, and pyroptotic macrophage death. The resulting inflammatory mediators, extracellular ASC specks, and secondary DAMP release amplify lung injury by recruiting and activating neutrophils, disrupting endothelial and epithelial barrier integrity, promoting capillary leak and alveolar edema, and impairing gas exchange. This process also generates feed-forward amplification, in which ongoing tissue injury releases additional danger signals that further sustain macrophage inflammasome activation.

## 3. Why Macrophage Inflammasomes Should Be Tamed Rather Than Abolished

Inflammasomes should be tamed rather than abolished. Canonical and non-canonical pathways activate caspase-1 or caspase-11/4/5, mature IL-1β/IL-18 and drive GSDMD-mediated pyroptosis [6,7,14,15,26]. In early infection, this can be useful: infected or stressed macrophages are sacrificed, intracellular microbes are exposed, and local myeloid recruitment is accelerated [6,26]. A blunt shutdown would therefore be biologically risky.

The risk is no longer theoretical. In a two-hit model of CLP followed by secondary *A. baumannii* pneumonia, post-septic mice failed to mount an adequate pulmonary inflammasome response. Lung NLRP3 and IL-1β induction was lower than in infected non-septic controls, caspase-1 activity in alveolar macrophages was weakened, and lung IL-1β fell by more than threefold. Even more striking, alveolar macrophages from post-septic mice were almost unable to release IL-1β after ex vivo LPS + nigericin stimulation [27]. Too little inflammasome activity can leave the lung undefended.

Conversely, IL-18/caspase-1 signaling, pyroptosis and extracellular ASC specks amplify leukocyte recruitment, permeability and edema [13,16]. In this setting, the inflammasome is no longer just an alarm. It becomes an amplifier. Selective output control may retain more host defense: necrosulfonamide inhibits GSDMD pore formation and inflammatory release without preventing ASC specks or TLR signaling, and improves survival in LPS sepsis [28]. This is a clean illustration of “taming”: the sensing apparatus remains, but the lytic inflammatory output is restrained.

Newer septic lung studies add more adjustable nodes. Ubiquitin-specific peptidase 48 (USP48) is increased in CLP-injured lungs and LPS-stimulated alveolar macrophages. Knocking it down reduces lung injury scores, Bronchoalveolar lavage fluid (BALF) protein, wet-to-dry ratio, airway resistance, BALF TNF-α/IL-1β/IL-6, F4/80 + TUNEL+ cell death, and caspase-1/PI double-positive alveolar macrophages [29]. Smurf1 acts in the opposite direction. Macrophage-targeted Smurf1 supplementation improves both endotoxin and CLP sepsis outcomes [30]. These studies make the same point from two angles: inflammasome activity is tunable.

Metabolism also tunes this axis. Liu et al. showed that pyroptotic macrophages shift toward aerobic glycolysis, accumulate lactate, and use lactate to promote NLRP3 lactylation. Lactate enhanced ASC recruitment, caspase-1 activation, GSDMD cleavage, N-GSDMD oligomerization, and IL-1β release; inhibition or knockdown of LDHA reduced lactate, impaired pyroptosis, and attenuated inflammatory responses in CLP sepsis [31]. Meanwhile, roburic acid offers a multi-target example: it binds NLRP3 and NCF1, suppresses pyroptosis and ROS-linked ferroptotic stress, and RBA-loaded nanoparticles reduce lung injury scores, wet/dry ratios, inflammatory cytokines, neutrophil infiltration, and multiorgan injury in septic mice [18]. The therapeutic message is not “block one molecule forever.” It is to interrupt pathological amplification loops at the right level.

This nuance is critical because sepsis is immunologically heterogeneous. Hyperinflammation, immune paralysis, endothelial injury, coagulopathy, metabolic stress, and organ-specific inflammation may coexist [9,32,33]. Clinical phenotyping and transcriptomic endotyping have repeatedly identified sepsis subgroups with different outcomes and potential treatment interactions [34,35,36,37]. The IL-1 blockade experience fits this logic. Anakinra is not standard therapy for all sepsis, but in a Macrophage activation syndrome-like (MAS-like) subgroup with hepatobiliary dysfunction and Disseminated intravascular coagulation (DIC), reanalysis of a phase III trial showed lower 28-day mortality with anakinra than placebo, approximately 35% vs. 65% [38]. Biology selected the responders.

**Figure 3:**
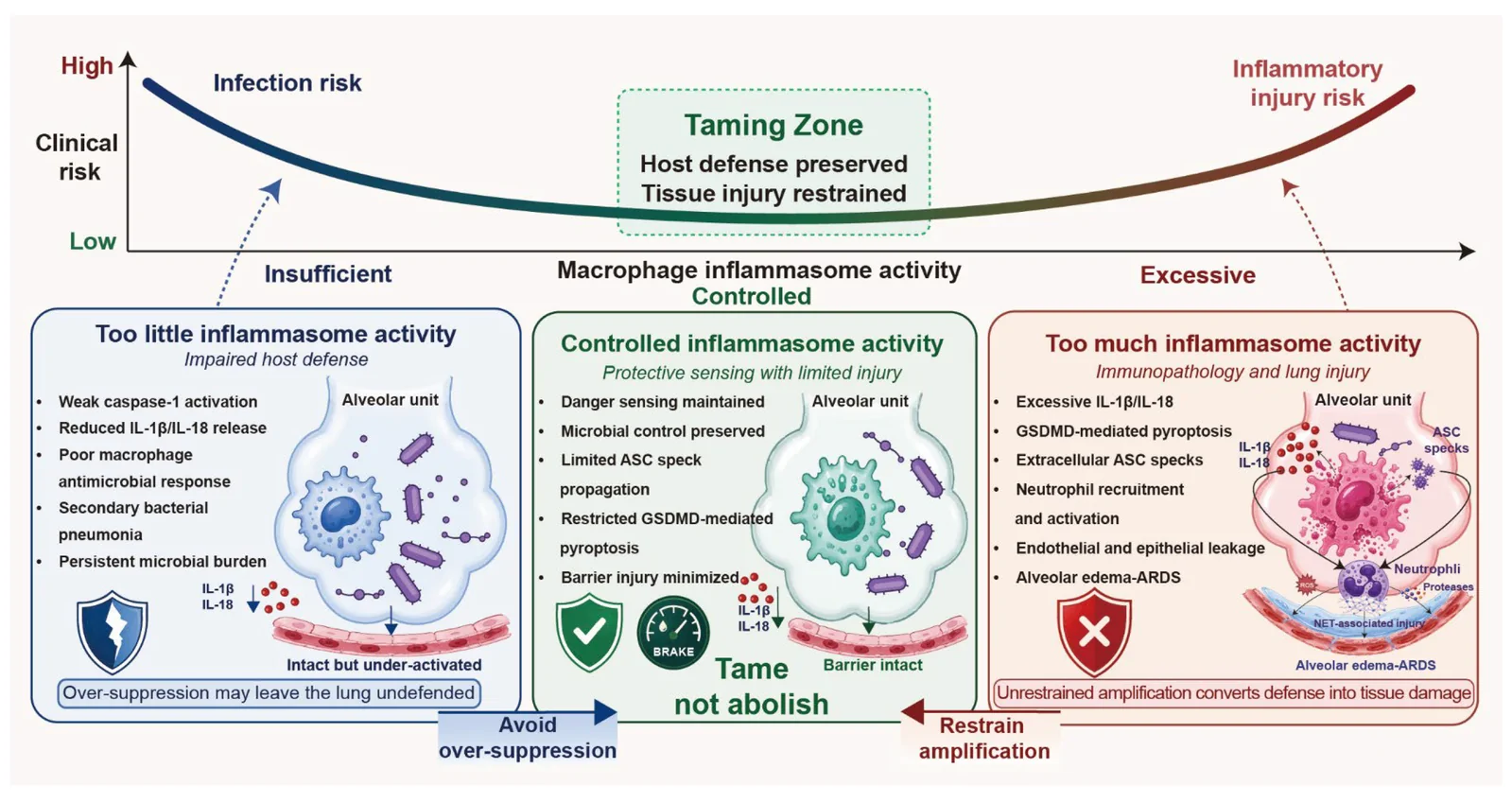
Conceptual “taming zone” for macrophage inflammasome activity in septic lung injury.

Thus, macrophage inflammasomes should be tamed, not abolished (Figure 3). We use the term “taming zone” to describe a functional therapeutic window in which inflammasome-mediated tissue injury is reduced without a meaningful loss of antimicrobial competence [39,40]. Experimentally, this window should be defined along two parallel axes. The first is suppression of pathological inflammatory output, assessed by measures such as IL-1β/IL-18 release, ASC speck formation, GSDMD cleavage, pyroptotic cell death, pulmonary barrier leakage, histological injury, and oxygenation [7,8,16,18,28,29,31,41]. The second is preservation of host defense, assessed by bacterial burden and dissemination, macrophage and neutrophil antimicrobial function, susceptibility to secondary infection, and survival [25,42,43]. An intervention would fall within the proposed taming zone only when improvement in the first axis is achieved without unacceptable deterioration in the second. Clinically, an analogous window would require longitudinal assessment of inflammasome activity, lung-injury trajectory, infection control, and immune competence rather than reliance on a single inflammatory biomarker. Importantly, the taming zone is currently a conceptual functional window rather than a validated quantitative range, and no clinical cutoff has yet been established to define it.

The aim is to preserve enough activity for pathogen sensing and early antimicrobial defense while limiting ASC speck propagation, GSDMD pore formation, metabolic overdrive, and “inflammasome-high” macrophage states that damage the lung. This framing aligns with disease tolerance: rather than only reducing pathogen burden, the host limits tissue injury at a given microbial load. Septic lung injury needs that kind of restraint. Inflammasomes are dangerous allies—too useful to eliminate, too destructive to ignore.

Both insufficient and excessive macrophage inflammasome activity can be harmful during bacterial sepsis. Excessive activation can amplify IL-1β/IL-18 release, GSDMD-mediated pyroptosis, ASC speck propagation, neutrophilic inflammation, and pulmonary barrier injury, whereas excessive suppression may compromise antimicrobial defense and increase susceptibility to persistent or secondary infection. The proposed “taming zone” represents a functional therapeutic window in which inflammatory tissue injury is reduced while antimicrobial competence remains preserved. Experimentally, its boundaries should therefore be determined by paired assessment of inflammatory and organ-injury endpoints together with bacterial clearance, dissemination, antimicrobial cell function, and survival. The zone is conceptual and does not currently represent a validated biomarker threshold or quantitative clinical range.

## 4. Lessons from Bats: Natural Restraint of Inflammasome-Driven Inflammation

Bats are often introduced as viral reservoirs, but that label is only the starting point. They host a wide range of viruses, including coronaviruses, henipaviruses, filoviruses and other zoonotic pathogens [44,45,46,47,48]. The more useful immunological question is why many of these infections remain clinically quiet in bats while related viruses cause severe inflammatory disease in spillover hosts. This is not because bats are immunologically inert. A recent Bat1K analysis of 115 mammalian genomes found that signatures of positive selection in immune genes are more prevalent in bats than in other mammalian orders; the ancestral chiropteran branch contained 42 immune genes under selection, almost twice the 22 expected by the regression model [49]. The same study reported that 17.8% of viral sequences detected in bats were coronaviruses, compared with only 1.4% in rodents, with even higher proportions in Rhinolophidae and Hipposideridae [49]. Bats, then, are exposed, immunologically adapted, and still unusually tolerant. That combination is what makes them interesting for septic lung injury.

Several comparative studies have converged on a similar idea: antiviral readiness is preserved, whereas inflammatory execution is restrained. High-quality bat genomes have revealed selection in immune, DNA damage response and antiviral pathways, supporting the view that flight, longevity and viral exposure shaped bat immunity together [50,51]. In *Pteropus alecto*, the type I IFN locus is contracted, yet IFN-α shows unusual constitutive expression, suggesting a basal antiviral state without the need for repeated inflammatory ignition [52]. Egyptian rousette bat genomics also revealed unexpected antiviral features, again pointing to intact defense but altered inflammatory wiring [53]. At the same time, bats have lost the Pyrin and HIN domain-containing (PYHIN) gene family across examined genomes, removing canonical AIM2-like DNA inflammasome sensing [54], and bat Stimulator of interferon genes (STING) carries sequence changes that dampen STING-dependent interferon activation [55]. Together, these observations illustrate substantial evolutionary remodeling of innate immune sensing in bats, but they should not be assumed to represent uniformly advantageous mechanisms that can be transferred directly to human disease.

The loss of PYHIN-family genes is an important example of this limitation. PYHIN proteins include AIM2, which links cytosolic DNA sensing to inflammasome activation and contributes to antimicrobial defense against several intracellular pathogens [56,57]. Consequently, attenuation or loss of AIM2-like sensing could reduce inflammatory responses to cytosolic DNA while simultaneously weakening pathogen-specific host defense. This trade-off is particularly relevant to bacterial sepsis, in which inflammasome activity may be required for pathogen containment in some cellular and infectious contexts. Thus, PYHIN loss is better viewed as evidence that inflammasome-associated sensing pathways can undergo substantial evolutionary remodeling in bats, rather than as a pathway that should itself be therapeutically reproduced in humans.

More broadly, this example illustrates an important boundary of the bat-inspired framework: evolutionary adaptations that are compatible with host defense in bats cannot be assumed to preserve antimicrobial competence when transferred individually to human sepsis. The translational value of bat biology therefore lies in identifying regulatory principles that separate inflammatory damage from host defense, not in reproducing every bat-specific immune alteration.

The clearest functional evidence for such selective inflammatory restraint comes from the NLRP3 inflammasome. Ahn and colleagues compared primary bat immune cells with human or mouse counterparts after LPS priming and ATP or nigericin stimulation [11]. The difference was not subtle. Human Peripheral blood mononuclear cells (PBMCs), mouse Bone marrow-derived macrophages (BMDMs) and Bone marrow-derived dendritic cells (BMDCs) formed ASC specks, released LDH, and secreted IL-1β after NLRP3 activation; bat PBMCs, BMDMs and BMDCs showed much weaker ASC speck formation, lower LDH release, and little IL-1β secretion. Importantly, this was not due to a globally silent TLR–NF-κB system. In bat BMDMs, TLR agonists could induce IL-1β, IL-6 and TNF-α transcripts by up to 115-, 67- and 57-fold, respectively, yet NLRP3 itself was poorly induced, reaching only about threefold even at higher stimulus doses. Bat BMDCs and PBMCs showed the same pattern, with NLRP3 rising only up to about fourfold and ninefold, respectively, despite preserved induction of other inflammatory genes [11]. This is a selective brake.

The brake also has a structural component. The same study identified multiple bat NLRP3 isoforms, including an exon 7-skipping variant within the leucine-rich repeat domain; in *P. alecto* tissues, exon 7-negative transcripts accounted for more than 60% of NLRP3 transcripts [11]. In reconstituted systems, bat NLRP3 isoforms were less able than human NLRP3 to drive ASC speck formation and IL-1β cleavage, and exon 7-negative isoforms were still weaker. Similar reduced NLRP3 activity was observed in *Myotis davidii*, a phylogenetically distant bat lineage. The implication is simple but important: bat NLRP3 is harder to push into full activation. This fits broader reviews of bat host defense, which emphasize that bats often separate pathogen control from inflammatory tissue damage rather than simply suppressing immunity [10,58].

Viral experiments sharpen the translational lesson. Infections with influenza A virus, Pteropine orthoreovirus 3, and MERS-CoV induced less NLRP3-mediated inflammation in bat cells, yet overall viral loads were not increased [11]. This finding sits at the center of the “lessons from bats” argument. It suggests that lowering inflammasome-mediated tissue injury does not necessarily mean abandoning pathogen control. That is precisely the balance missing in many forms of sepsis-associated lung injury.

Other bat studies push this idea downstream. Goh and colleagues examined DNA inflammasome signaling after the evolutionary loss of AIM2-like receptors in bats [59]. When human AIM2 was introduced into bat cells, DNA stimulation restored ASC speck formation; in *P. alecto* kidney cells, poly (dA) induced dose-dependent ASC specks and AIM2 oligomerization, with approximately 12-fold and 15-fold increases at 2.0 μg/mL, respectively. Yet this restored platform did not translate into normal effector output. AIM2-reconstituted bat BMDMs failed to produce detectable mature IL-1β, showed minimal LDH release, and had weak caspase-1 activity by Fluorescent-labeled inhibitor of caspases (FLICA) assay. The authors traced this to lineage-specific downstream restraints: two positively selected residues in *P. alecto* caspase-1 impaired enzymatic activity, whereas *M. davidii* showed altered IL-1β cleavage-site biology [59]. Bats have not installed one brake. They have installed several.

Bat ASC2 provides the most direct link to therapeutic thinking. ASC is the shared adaptor for several inflammasomes, and ASC specks can propagate inflammation beyond the initially activated cell [16,60]. ASC2 was found in all 13 examined bat species, contrary to earlier assumptions that ASC2 was primate-restricted [12]. In bat splenocyte subsets, ASC2 mRNA was higher than ASC in putative monocytes/macrophages and similar to ASC in dendritic cells, while human monocytes/macrophages and dendritic cells showed no or minimal ASC2 expression. At the protein level, bat ASC2 was higher in CD11b+ myeloid cells than in CD11b− leukocytes [12]. Functionally, bat ASC2 interacted with ASC, formed filament-like structures, disrupted ASC speck formation, and inhibited human NLRP3 and AIM2 inflammasomes more potently than human ASC2. In THP-1 macrophages, bat ASC2 reduced nigericin-triggered LDH release and IL-1β secretion, and it also suppressed AIM2-driven responses after poly (dA) transfection. In keratinocytes, it inhibited NLRP1 activation by talabostat [12]. That breadth matters.

The mouse data make bat ASC2 harder to dismiss as a cell-culture curiosity. In BMDMs from doxycycline-inducible bat ASC2 transgenic mice, bat ASC2 reduced cell death and IL-1β secretion after NLRP3, AIM2 and NLRC4 stimulation [12]. In vivo, Monosodium urate (MSU) crystal-induced sterile peritonitis produced neutrophil and inflammatory monocyte recruitment in WT mice, but these responses and peritoneal IL-1β were reduced in bat ASC2-expressing mice. ASC speck-induced peritonitis was also blunted, supporting an ASC-level mechanism. In H1N1 influenza infection, all WT mice died by 14 days, whereas mortality was about 50% in bat ASC2-expressing mice; lung IL-1β was significantly reduced, while viral loads were similar [12]. TAT-delivered bat ASC2 further suppressed human NLRP3 activation in a dose-dependent manner and reduced IL-1β production by primed human monocytes exposed to SARS-CoV-2 immune complexes [12]. A Nature Reviews Immunology research highlight summarized this work as evidence that bat ASC2 can negatively regulate inflammasome activation across sterile and infectious settings [61]. This is probably the strongest proof-of-concept that a bat-derived inflammasome regulator can restrain inflammation without obviously collapsing antiviral control.

More recent in vivo single-cell work gives a complementary view of bat viral tolerance. In male Jamaican fruit bats infected with H18N11 influenza A virus, investigators profiled 33,974 intestinal cells and 20,600 mesenteric cells [62]. Infectious virus was detected in rectal swabs from all WT H18N11-infected bats up to 7 days post-infection, yet transcriptional inflammation was modest: most cell types did not show significant induction of interferons or pro-inflammatory cytokines, while ISGs such as *Ifi6*, *Ly6e*, *Mx1* and *Isg15* were moderately induced at 3 days post-infection in epithelial and immune cell compartments [62]. H18N11 infection was mapped largely to leukocytes, including macrophages, B cells and NK/T cells. Here again, viral infection and immune-cell tropism existed, but the inflammatory tone remained contained.

Bat inflammatory restraint may also extend beyond classical innate signaling. Long-lived bats show coordinated adaptations in DNA repair, proteostasis, mitochondrial quality control and inflammatory dampening, offering a broader framework for damage tolerance rather than simple immune suppression [63,64,65]. Recent work on bat antibodies adds another layer: at temperatures typical of active flight, bat IgG broadened antigen-binding diversity, including recognition of pathogen-derived antigens and damaged epithelial or endothelial cells, whereas human IgG did not show comparable temperature sensitivity [66]. This does not directly prove inflammasome control, but it supports a larger principle: bat immunity may be dynamically tuned to physiological state, pathogen exposure and tissue damage.

An important limitation of this evidence base is its concentration within a relatively small number of experimental studies. In particular, much of the direct functional evidence for attenuated NLRP3 responsiveness and ASC2-mediated inflammasome suppression derives from studies led by the same research group [11,12]. Broader comparative genomic and immunological studies from multiple groups support substantial remodeling of innate immune pathways in bats, including changes in interferon-associated and cytosolic nucleic-acid-sensing pathways [49,50,51,52,53,55]. However, these studies address related features of bat immunity rather than independently replicating the specific NLRP3- or ASC2-centered mechanisms reported in [11,12]. Additional work has identified regulation at the caspase-1/IL-1β level [59], providing further support for the broader concept of restrained inflammatory output, but again does not constitute direct replication of the NLRP3 or ASC2 findings. The latter mechanisms should therefore be regarded as compelling but incompletely replicated observations rather than universally established features across bat species. Their conservation across species, tissues, infectious contexts, and independent experimental systems remains to be determined.

Within these limitations, bat studies support a distinctive biological principle: robust immune surveillance does not necessarily require unrestricted inflammasome execution. Attenuated NLRP3 responsiveness, remodeling of DNA-sensing pathways, and ASC2-mediated suppression of ASC-dependent inflammasomes provide complementary examples in which inflammatory output is constrained rather than simply eliminated. Nevertheless, these observations should not be interpreted as a ready-made therapeutic blueprint for sepsis. Much of the functional evidence has been generated in viral infection or sterile inflammatory settings, and direct evidence that these adaptations preserve host defense during bacterial or polymicrobial sepsis remains limited. Bat biology therefore establishes the biological plausibility of restrained inflammatory execution, but not yet its therapeutic validity in bacterial sepsis.

This distinction is critical because bacterial sepsis imposes a different constraint: limiting inflammatory tissue injury is beneficial only if antimicrobial competence is retained. Inflammasome signaling itself participates in both processes, and its contribution may change with the pathogen, route of infection, disease stage, and cellular compartment involved. Thus, before asking how bat-associated mechanisms might be therapeutically reproduced, a more immediate question must be addressed: does experimental bacterial infection support the possibility that inflammasome activity can be restrained without sacrificing pathogen control? The available evidence does not provide a simple yes-or-no answer; instead, it reveals a strongly context-dependent balance between inflammatory injury and antimicrobial defense.

## 5. Bridging Bat Inflammasome Biology to Bacterial Sepsis

The key translational issue is therefore not whether inflammasome signaling is beneficial or harmful in bacterial sepsis as a whole, but when, where, and through which cellular compartment its activity becomes maladaptive. Gram-negative infection also introduces an important pathway that is incompletely represented by most bat studies: cytosolic LPS sensing through caspase-4 and caspase-5 in humans and caspase-11 in mice. Activation of this non-canonical inflammasome pathway promotes GSDMD-mediated membrane permeabilization and can secondarily engage NLRP3 and caspase-1 [67,68,69]. Its biological consequences, however, vary considerably across experimental settings.

This model dependence is evident in pulmonary *Klebsiella pneumoniae* infection. In the gradually evolving airway-initiated pneumosepsis model used by Perlee et al., pulmonary caspase-11 expression increased during infection, and Casp11-deficient mice carried higher lung bacterial burdens at both 12 and 48 h [42]. Importantly, impaired bacterial control was not accompanied by a broad reduction in pulmonary inflammation or injury. Cleaved GSDMD was undetectable in lung homogenates, and caspase-11 deficiency did not significantly alter histological lung pathology, Ly6G-positive lung area, pulmonary Myeloperoxidase (MPO) concentrations, BALF neutrophil numbers, or BALF protein concentrations [42]. Lung IL-1α and IL-1β concentrations were also comparable between genotypes at both time points, although BALF IL-1α was lower and lung TNF higher in Casp11-deficient mice at 48 h. Conversely, cross-linked fibrin and D-dimer formation were reduced in Casp11-deficient lungs, suggesting that caspase-11-dependent local coagulation may contribute to pulmonary host defense rather than merely marking inflammatory damage. In this setting, therefore, removing caspase-11 impaired bacterial containment without producing the corresponding global improvement in lung inflammation that might be expected from simple pathway inhibition.

The balance becomes still more informative when caspase-11 is manipulated in defined cellular compartments. Kumari et al. challenged conditional knockout mice with 20 mg/kg LPS and found that deletion of Casp11 in LyzM+ monocytes/macrophages significantly prolonged survival, whereas neutrophil-specific deletion did not confer protection; deletion in CD11c^+^ cells produced a weaker phenotype [25]. Monocyte/macrophage-specific deletion also reduced circulating IL-18 and IL-1β, attenuated the release of the DAMPs IL-1α, HMGB1, and galectin-1, and reduced GSDMD cleavage in the spleen and liver. In contrast, TLR4-dependent IFN-β, TNF, and IL-6 responses were largely preserved. By 16 h, these mice also showed less liver degeneration and necrosis and lower circulating AST and LDH. These findings locate a substantial component of caspase-11-mediated endotoxin injury within the monocyte/macrophage compartment rather than reflecting uniform suppression of the inflammatory response.

The same cellular pathway, however, became protective when the challenge changed from endotoxin to replicating bacteria. Following intraperitoneal infection with 10^3^ CFU *Burkholderia thailandensis*, all control Casp11^fl/fl^ mice survived the 30-day observation period, whereas global Casp11-deficient animals died within 4–5 days [25]. Mice lacking Casp11 in LyzM^+^ cells died within 5–7 days and those with neutrophil-specific deletion within 7–11 days, while CD11c^+^ cell- and intestinal epithelial cell-specific knockouts survived similarly to controls. Three days after infection, bacterial loads in the spleen and liver were undetectable in control animals but remained high in global Casp11-deficient mice; loss of Casp11 in LyzM^+^ cells or neutrophils likewise compromised bacterial control. Thus, a pathway that substantially contributed to macrophage-associated injury during endotoxemia was required in the same broad myeloid compartment for effective defense against intracellular Gram-negative infection.

Even within *K. pneumoniae* infection, however, the direction of the phenotype is not fixed. In a recent systemic infection model, intraperitoneal *K. pneumoniae* challenge reduced arterial oxygen saturation from approximately 95% to 70% within 7 h, whereas 10 mg/kg purified LPS did not reproduce this early hypoxemia [43]. All wild-type mice receiving 10^6^ CFU died within 20 h, while deletion of *Casp11* or *Nlrp3* significantly prolonged survival; disruption of *Tlr4* or combined *Gsdmd/Gsdme* did not produce the same benefit. *Casp11-* and *Nlrp3-*deficient animals also maintained better oxygenation and showed lower pulmonary bacterial burdens, reduced lung IL-6 and MPO activity, and less prominent neutrophil infiltration and alveolar-wall thickening.

The macrophage experiments in the same study provide a possible functional explanation. Depletion of macrophages before *K. pneumoniae* challenge shortened survival and increased bacterial burdens in multiple organs. Yet *Casp11-* or *Nlrp3-*deficient bone-marrow-derived macrophages internalized similar numbers of bacteria as wild-type cells at 90 min but contained fewer viable intracellular bacteria at 6 h. This enhanced killing coincided with greater mitochondrial ROS production and occurred without a corresponding increase in LDH-defined cell death. Moreover, the bacterial-clearance advantage disappeared after treatment with either the ROS scavenger NAC or the mitochondrial ROS-targeted antioxidant MitoQ, and MitoQ pretreatment increased pulmonary bacterial burden in vivo [43]. Thus, reduced caspase-11/NLRP3 signaling did not merely attenuate inflammation in this model; it altered macrophage antimicrobial function through an mtROS-dependent mechanism.

These apparently divergent results should not be forced into a single direction. Instead, they define the boundary of the bat-to-sepsis analogy. Bat studies suggest that inflammatory execution can be restrained without necessarily abolishing immune competence, whereas bacterial studies show that achieving such separation is highly dependent on the infection model, cellular compartment, and antimicrobial function being measured. In airway-initiated pneumosepsis, loss of caspase-11 impaired bacterial containment without substantially alleviating pulmonary inflammation; during *B. thailandensis* infection, myeloid caspase-11 was indispensable for bacterial clearance and survival; yet in systemic *K. pneumoniae* infection, loss of caspase-11 or NLRP3 improved both lung injury and bacterial control. The relevant therapeutic problem is therefore not whether an inflammasome pathway is intrinsically “good” or “bad”, but which component should be restrained, in which cell population, during which phase of infection, and to what extent.

This distinction also refines what should be considered “bat-inspired”. The goal is not to reproduce a generalized low-inflammatory bat phenotype in septic patients. Rather, bat biology provides proof-of-principle that inflammatory execution and effective host defense can, under some biological conditions, be partially uncoupled. The bacterial studies above indicate how difficult this separation may be to reproduce therapeutically. A candidate intervention should therefore satisfy a more stringent standard than reduction in IL-1β or improved survival after endotoxin challenge: it should attenuate damaging inflammasome outputs while preserving macrophage- and neutrophil-dependent pathogen containment. Ideally, these effects should be demonstrated simultaneously within the same experimental system by measuring tissue injury and organ function alongside bacterial burden, dissemination, secondary infection susceptibility, and survival.

The translational problem therefore shifts from inflammasome suppression to selective inflammasome control. This provides the basis for evaluating the therapeutic approaches discussed below.

## 6. Translating Inflammasome Restraint into Therapeutic Strategies

The context-dependent effects described above provide a framework for considering therapeutic modulation of inflammasomes in septic lung injury. Several points along the inflammasome pathway are potentially amenable to intervention, including NLRP3 activation, ASC-dependent assembly, downstream GSDMD-mediated execution, and the metabolic and quality-control pathways that shape macrophage responses. Among these, ASC2 represents a direct molecular lead from bat studies, whereas most other approaches were developed independently and are considered here in light of the broader principle of restrained inflammasome activation. Accordingly, we distinguish throughout this section among mechanisms directly demonstrated in bats, therapeutic principles supported by bat biology but tested using non-bat experimental systems, and translational strategies that remain hypothetical.

The distinctive contribution of the bat literature is therefore not the identification of inflammasome inhibition as a therapeutic concept, but the generation of more specific hypotheses about how inflammatory restraint might be achieved without globally disabling innate immunity. In particular, bat studies suggest that reduced inducibility of NLRP3 can coexist with preserved TLR-responsive inflammatory transcription [11], and that an endogenous ASC-interacting protein can suppress ASC-dependent inflammasome assembly without simply eliminating upstream pathogen sensing [12]. These observations generate two testable translational predictions: first, that partial or state-dependent reduction in inflammasome responsiveness may be sufficient to reduce inflammatory injury without requiring complete pathway ablation; and second, that endogenous regulatory interfaces such as ASC–ASC2 interactions may provide intervention points distinct from conventional inhibition of inflammasome sensors or cytokines. Whether either prediction remains valid during bacterial sepsis is unknown and requires direct experimental testing.

The following sections examine these strategies together with the cellular context and immune state that may influence their effects.

### 6.1. NLRP3 Threshold Modulation

NLRP3 is an attractive upstream target because it integrates multiple signals relevant to septic lung injury, including extracellular ATP, mitochondrial dysfunction, ROS, ionic disturbance, and tissue-derived danger signals. Bat NLRP3 priming and activation are selectively dampened while other inflammatory responses remain inducible [11,59], suggesting that modulation of NLRP3 activity does not necessarily require broad suppression of innate immune signaling.

MCC950 showed that selective NLRP3 inhibition is pharmacologically feasible: it inhibited ATP-induced IL-1β release with IC50 values of approximately 7.5 nM in mouse BMDMs and 8.1 nM in human monocyte-derived macrophages, while largely preserving LPS-induced TNF-α [70]. It also blocked NLRP3 responses to nigericin and MSU but did not inhibit AIM2, NLRC4, NLRP1, TLR signaling, or NLRP3 priming [70].

However, pharmacological selectivity should be distinguished from translational maturity. MCC950 has demonstrated in vivo efficacy across multiple experimental inflammatory disease models [7], but its relevance to septic lung injury remains preclinical and should not be interpreted as evidence of established clinical efficacy. More broadly, the agents discussed in this review span different levels of evidence: some have shown therapeutic effects in animal models of acute lung injury or sepsis, whereas others currently provide primarily mechanistic or proof-of-concept evidence in cellular or experimental systems. We therefore distinguish in vivo disease-model efficacy from mechanistic proof-of-concept throughout the following discussion.

Pharmacological modulation of this pathway spans different levels of translational maturity. MCC950 (CRID3) is a potent and selective direct NLRP3 inhibitor that has shown efficacy across numerous preclinical inflammatory disease models, but its clinical development was limited by pharmacokinetic and safety concerns, including hepatotoxicity, and it should therefore be regarded primarily as a benchmark experimental NLRP3 inhibitor rather than an established therapeutic agent [71]. Dapansutrile (OLT1177), another orally active selective NLRP3 inhibitor, has progressed further into human testing. In an open-label phase 2a proof-of-concept study involving 34 patients with acute gout, dapansutrile was administered at doses of 100–2000 mg/day and showed reductions in joint pain with an acceptable short-term safety profile; however, these findings were obtained outside sepsis and do not establish efficacy in septic lung injury [72].

Downstream blockade of IL-1 signaling provides a clinically more advanced, although less NLRP3-specific, approach. Anakinra blocks the IL-1 receptor rather than NLRP3 itself. In a post hoc reanalysis of a previous phase III severe-sepsis trial [38], 43 of 763 evaluable patients had concurrent hepatobiliary dysfunction and disseminated intravascular coagulation as macrophage activation syndrome-like features. In this small subgroup, 28-day survival was 65.4% with anakinra versus 35.3% with placebo, corresponding to a hazard ratio for death of 0.28 (95% CI, 0.11–0.71; *p* = 0.0071). No survival difference was observed in patients without this phenotype. These findings are therefore hypothesis-generating rather than confirmatory and support biomarker-guided evaluation rather than indiscriminate IL-1 blockade in sepsis.

More recently, the ImmunoSep randomized trial further supported a precision-immunotherapy framework [73]: patients classified as having macrophage activation-like syndrome received anakinra, whereas those with sepsis-induced immunoparalysis received interferon-γ. Precision immunotherapy improved the prespecified organ-dysfunction endpoint compared with placebo, although 28-day mortality was not significantly different, reinforcing the need to match immune-directed therapy to host-response phenotype rather than applying uniform inflammatory suppression.

### 6.2. ASC-Centered Restraint

ASC provides a distinct intervention point because it serves as a common adaptor for several inflammasomes and can propagate inflammatory signaling through extracellular ASC specks [16,60]. Bat ASC2 is particularly relevant in this context because it directly interferes with ASC-dependent inflammasome assembly. It interacts with ASC, disrupts ASC speck formation, suppresses NLRP3, AIM2 and NLRC4 inflammasomes, reduces MSU- and ASC speck-induced peritonitis, and improves survival after H1N1 infection while viral loads remain similar [12]. These experimentally demonstrated effects provide the rationale for, but should be distinguished from, proposed therapeutic applications. Potential future strategies might include ASC2-derived inhibitory peptides, engineered ASC2-like constructs, transient inhibitors of ASC polymerization, or macrophage-directed delivery systems. These approaches remain hypothetical and have not yet been validated as therapies for bacterial sepsis.

For septic lung injury, this matters because multiple inflammasome sensors may be activated simultaneously by PAMPs, DAMPs, mitochondrial stress, extracellular ATP and tissue injury. Targeting NLRP3 alone may miss AIM2-, NLRC4- or non-canonical inflammasome inputs. An ASC-level brake could, at least in principle, restrain the shared speck-forming amplifier. The caution is that ASC is a shared adaptor for multiple inflammasomes, making the degree, duration, and cellular distribution of its inhibition particularly important. To our knowledge, ASC2-based modulation has not yet been evaluated in polymicrobial sepsis or bacterial septic lung injury. Whether ASC2-mediated restraint can reduce tissue-damaging inflammasome activity while preserving antimicrobial defense therefore remains unknown and will require direct testing in clinically relevant bacterial sepsis models.

### 6.3. Downstream Control of Pyroptotic Execution

Targeting downstream inflammatory execution offers a different strategy from inhibiting inflammasome assembly itself. GSDMD is particularly attractive because pore formation lies downstream of inflammatory sensing and directly contributes to pyroptotic membrane rupture and inflammatory mediator release.

Necrosulfonamide and disulfiram can inhibit GSDMD pore formation and reduce inflammatory cell death in experimental systems [14,28,41]. However, downstream blockade should not be assumed to reproduce the effects of upstream inflammasome inhibition. In the systemic *K. pneumoniae* model discussed above, combined *Gsdmd*/*Gsdme* deficiency did not reproduce the survival benefit observed after *Casp11* or *Nlrp3* deletion [43].

IL-1 blockade provides another means of limiting downstream inflammatory signaling, although it has not shown consistent benefit in unselected sepsis populations. In a post hoc reanalysis of a randomized trial, 43 of 763 evaluable patients had concurrent hepatobiliary dysfunction and disseminated intravascular coagulation, a phenotype used as a clinical surrogate for macrophage activation syndrome. Within this small subgroup, 28-day survival was 65.4% with anakinra versus 35.3% with placebo, corresponding to a hazard ratio for death of 0.28 (95% CI, 0.11–0.71; *p* = 0.0071) [38]. Because the subgroup was defined retrospectively and represented only a small fraction of the original trial population, this finding should be considered hypothesis-generating rather than evidence for an established sepsis indication for IL-1 blockade. Prospective validation is required before this phenotype can be used for treatment selection. Another experimental approach is modulation of the NLRP3–NEK7 interaction: 4-hydroxynonenal reduces ASC speck formation, caspase-1 cleavage, GSDMD cleavage, and IL-1β maturation without suppressing AIM2 or NLRC4 inflammasomes [74].

### 6.4. Macrophage State, Autophagy, and Metabolic Context

Septic lungs contain resident alveolar macrophages, recruited monocyte-derived macrophages, inflammatory monocytes, interstitial macrophages and chemokine-producing macrophage states [17,75,76,77]. These populations are not interchangeable. Single-cell studies suggest that selected inflammatory monocyte/macrophage subsets, rather than all macrophages, are most closely linked to septic lung injury phenotypes [17,75]. This cellular heterogeneity is particularly relevant to interventions that alter inflammasome activity, autophagy, mitochondrial quality control, or cellular metabolism.

Cellular origin provides an additional layer of heterogeneity. Resident alveolar macrophages are shaped by long-term adaptation to the alveolar niche, whereas recruited monocyte-derived macrophages enter the injured lung from the circulation and can retain distinct transcriptional and functional programs even after occupying the same airspace [76,77]. These differences may influence therapeutic response because an intervention that suppresses inflammasome signaling or alters cellular metabolism in recruited inflammatory macrophages may not produce the same effect in niche-adapted resident alveolar macrophages. Conversely, broadly targeting both populations could suppress a disease-promoting recruited macrophage states while simultaneously perturbing homeostatic functions of resident macrophages. Therapeutic responses should therefore be evaluated separately in resident and recruited macrophage compartments rather than inferred from bulk lung macrophages or a single ex vivo macrophage model.

Metabolism and redox biology provide a particularly clear example of this context dependence. Roburic acid targets both NLRP3 and Neutrophil cytosolic factor 1 (NCF1), reducing ROS-linked pyroptosis/ferroptosis crosstalk and septic lung injury [18]. The importance of macrophage origin is illustrated by the paradoxical effects of itaconate. Although native itaconate has anti-inflammatory effects in BMDMs, it can produce the opposite response in tissue-resident alveolar macrophages, increasing IL-6, IL-12 and mature IL-1β production, ASC speck formation, and caspase-1/GSDMD activation while aggravating LPS-induced ALI in vivo [78]. This divergence is more than a technical discrepancy between experimental systems: it demonstrates that the direction of a metabolic intervention can change with macrophage identity and tissue niche. Findings obtained in BMDMs should therefore not be assumed to predict the response of resident alveolar macrophages, particularly when metabolic pathways are being considered as therapeutic targets in septic lung injury.

Beyond cellular metabolism, endogenous quality-control pathways provide another means of modulating macrophage inflammasome activity. In CLP-induced ALI, SIRT1 loss disrupts *Rab7*-dependent mitophagy, allowing damaged mitochondria, mtROS and cytosolic mtDNA to activate NLRP3 and STING; restoring this axis improves inflammation and barrier injury [79]. In macrophages, Tripartite motif containing 21 (TRIM21) promotes K11-linked ubiquitination and degradation of Unc-51 like autophagy activating kinase 1 (ULK1), Sequestosome 1 (SQSTM1)/p62, Beclin 1 (BECN1) and Microtubule-associated protein 1 light chain 3 beta (LC3B), thereby impairing autophagy; macrophage-specific *Trim21* deletion reduces mortality, lung injury, vascular leakage, BALF protein and IL1B/IL6/IL18/TNF expression in septic ALI [80]. PV-K nanoparticles extend this into a delivery strategy: they enhance Nrf2–SQSTM1/p62 signaling, promote SQSTM1/p62–NLRP3 interaction and autolysosomal NLRP3 degradation, reduce GSDMD-N and protect against LPS- and CLP-induced ALI; this protection disappears in *Nrf2−/−* mice [81].

These intervention points differ substantially in both selectivity and biological risk. NLRP3 inhibition offers relatively pathway-selective upstream control but may be insufficient when AIM2, NLRC4, or non-canonical inflammasomes contribute substantially to disease [7]. ASC-centered intervention could restrain several ASC-dependent inflammasomes simultaneously, but this broader coverage may also increase the risk of impairing protective inflammasome functions [12,16,60]. Targeting GSDMD acts further downstream and may limit pyroptotic membrane injury while leaving upstream sensing intact [14,41], although downstream blockade does not necessarily reproduce the antimicrobial or survival effects of NLRP3 or caspase-11 modulation [43]. Autophagy and mitochondrial quality-control pathways may suppress inflammasome activation while simultaneously improving cellular homeostasis, but their pleiotropic effects make target specificity and dose–response relationships more difficult to predict [79,80,81]. Metabolic interventions offer the possibility of reprogramming inflammatory macrophage states rather than directly blocking inflammasome components, yet their effects are particularly dependent on macrophage origin and tissue niche, as illustrated by the divergent effects of itaconate in BMDMs and resident alveolar macrophages [78]. Thus, no intervention level can currently be considered universally preferable; the appropriate target is likely to depend on the dominant inflammasome pathway, macrophage state, infection context, and phase of sepsis.

### 6.5. Biomarker-Guided Patient Selection and Timing

The context dependence of inflammasome signaling makes patient selection and treatment timing central to clinical translation. Elevated systemic inflammation alone is unlikely to identify patients in whom inflammasome activity is a dominant and therapeutically modifiable process. A more informative strategy would combine evidence of inflammasome activation with the trajectory of lung injury and an assessment of concurrent immune competence. In practical terms, these dimensions should not be interpreted independently: evidence of active inflammasome signaling may support pathway-directed treatment only when accompanied by progressive inflammatory organ injury and without strong evidence of impaired antimicrobial immunity.

Identifying inflammasome-active macrophages in patients presents an additional challenge because transcriptional evidence of inflammasome components does not necessarily indicate functional inflammasome activation [82]. Single-cell RNA sequencing can resolve inflammatory monocyte/macrophage states and identify populations enriched for genes such as *NLRP3*, *CASP1*, *GSDMD*, and *IL1B*, but these measurements primarily reflect transcriptional potential rather than inflammasome assembly, proteolytic activation, or pyroptotic execution. A more rigorous research definition would therefore combine cell-state information with functional evidence, such as ASC speck formation, caspase-1 activation or GSDMD, and mature IL-1β/IL-18, ideally within the same macrophage population. In patients with lung injury, BALF-based flow cytometry or imaging approaches could potentially provide cell-resolved assessment when bronchoscopy is clinically indicated [83], whereas circulating IL-1β/IL-18, extracellular ASC specks, or related markers may offer less invasive but less cell-specific readouts [82,83,84]. At present, however, no macrophage-specific marker panel has been prospectively validated for routine identification of an inflammasome-active state in septic lung injury.

For prospective clinical studies, a practical biomarker framework could therefore combine three complementary dimensions rather than rely on a single marker: a readily measurable circulating inflammasome-related marker, such as IL-18 [13]; a more direct indicator of inflammasome assembly or execution, such as ASC specks or activated caspase-1/GSDMD when technically feasible [16,82,83,84]; and a parallel measure of immune competence, such as mHLA-DR [85]. These measurements should be interpreted together with the trajectory of pulmonary dysfunction and microbiological control. This framework is intended as a research strategy rather than a diagnostic definition: the optimal marker combination, sampling compartment, timing, and cutoff values remain to be established prospectively. Accordingly, we consider “inflammasome-high” a provisional research phenotype rather than a validated clinical classification; prospective studies will be required to establish its assignment criteria, assay thresholds, reproducibility, and clinically acceptable turnaround time.

Sepsis-associated hyperinflammation and immunosuppression can coexist, and their relative contribution changes over time [8,9]. In a 20-year cohort of 1023 septic shock patients, mHLA-DR remained markedly depressed during the first ICU week; by day 5/7, 62% of patients were still below 8000 AB/C, and persistent low expression was associated with mortality and ICU-acquired infections [85]. Such measurements could help identify patients in whom additional inflammatory suppression may carry greater risk. Conversely, preserved or recovering immune competence should not by itself be interpreted as an indication for inflammasome inhibition; it would need to coincide with evidence of persistent inflammasome activity and ongoing inflammatory lung injury.

**Figure 4:**
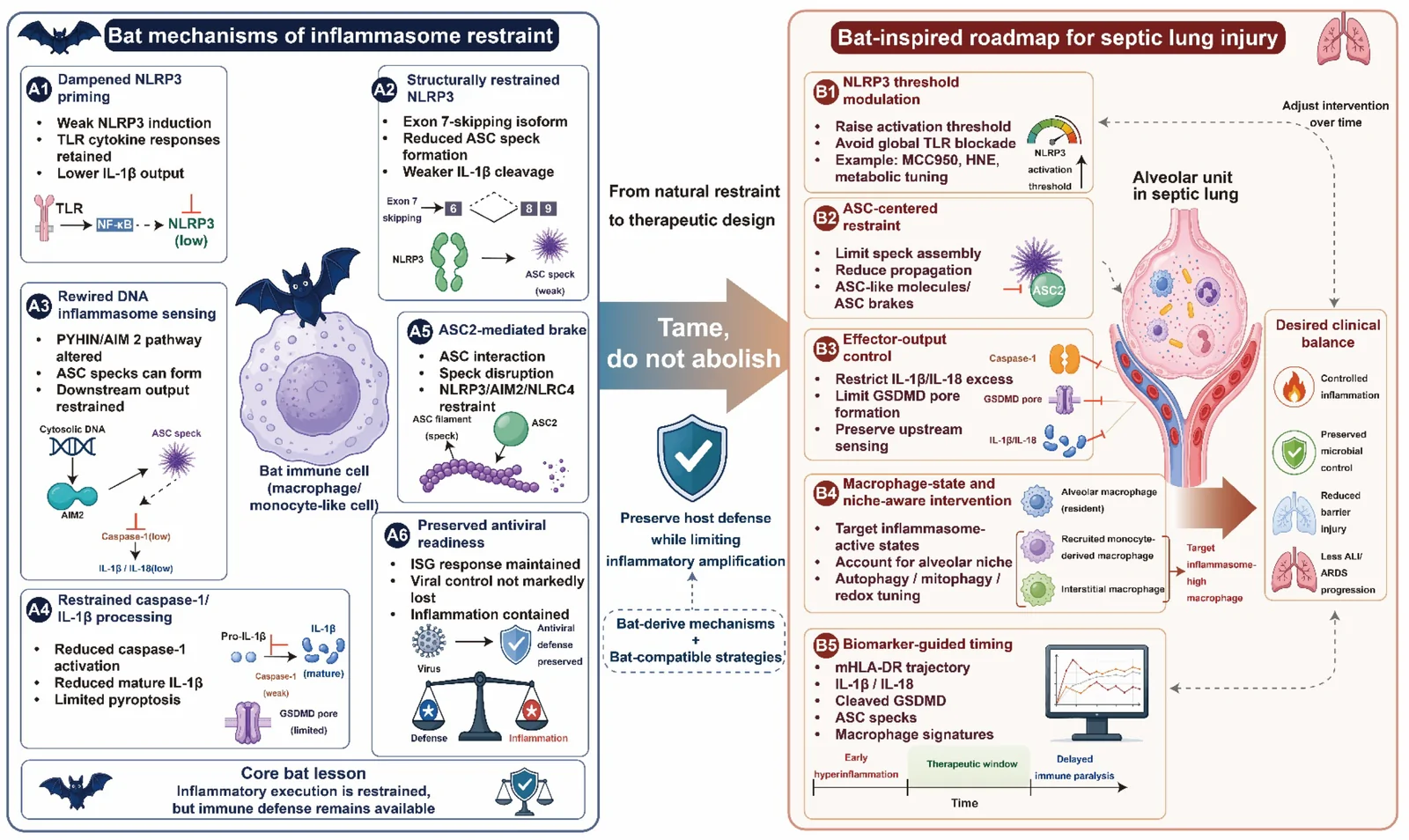
Bat inflammasome biology and potential routes toward therapeutic translation.

Prospective studies will therefore need to determine whether dynamic combinations of inflammasome activity, lung-injury progression, and immune-competence markers can identify patients in whom pathway-directed treatment reduces tissue injury without compromising antimicrobial defense (Figure 4).

Bats restrain inflammasome-mediated inflammatory execution through multiple mechanisms, including dampened NLRP3 responsiveness, altered DNA inflammasome sensing, reduced caspase-1/IL-1β processing, and ASC2-mediated inhibition of ASC-dependent inflammasomes, while maintaining antiviral readiness. These mechanisms support a bat-inspired therapeutic principle for septic lung injury: preserve host defense while limiting self-amplifying inflammatory damage. Potential translation requires NLRP3 threshold modulation, ASC-centered restraint, effector-output control, macrophage-state and niche-aware intervention, and biomarker-guided timing, with careful validation across species, infection contexts, tissue compartments, and immune phases.

### 6.6. Experimental Validation Strategy

The choice of experimental model is particularly important for evaluating inflammasome-directed interventions because endotoxemia, polymicrobial peritonitis, and pneumonia-derived sepsis impose different requirements on antimicrobial host defense. LPS challenge is useful for mechanistic dissection of inflammasome-mediated inflammatory injury but does not reproduce replicating infection and therefore cannot determine whether an intervention preserves bacterial clearance [25]. CLP provides a clinically relevant polymicrobial challenge and is valuable for assessing systemic inflammation, organ injury, bacterial dissemination, and survival [86], but it does not reproduce a primary pulmonary infectious focus. For interventions intended to prevent septic lung injury while preserving pulmonary host defense, we therefore consider bacterial pneumonia progressing to sepsis—such as an airway-initiated K. pneumoniae model—the most informative primary validation model [42], complemented by CLP to test whether the effect is preserved in polymicrobial sepsis [86]. Endotoxemia should be retained primarily as a mechanistic model rather than used alone to establish therapeutic efficacy.

Importantly, validation should assess both sides of the proposed “taming zone” within the same experiment. Lung injury and inflammasome activity should be measured together with pulmonary and systemic bacterial burden, dissemination, macrophage and neutrophil antimicrobial function, organ dysfunction, and survival [25,42,43]. Where possible, resident alveolar macrophages and recruited monocyte-derived macrophages should be analyzed separately. Such a design would determine not only whether an intervention suppresses inflammatory injury, but whether it does so without compromising the host-defense functions that are absent from sterile endotoxemia models. Beyond demonstrating preserved host defense in experimental infection, a second requirement is to establish that this balance persists when inflammasome modulation is incorporated into standard sepsis treatment.

Any inflammasome-directed intervention in bacterial sepsis should be developed as an adjunct to, rather than a substitute for, antimicrobial therapy, source control, and organ-supportive care. Importantly, its effects should be evaluated under conditions that approximate clinical treatment, including appropriate antimicrobial therapy initiated after establishment of infection. This is particularly relevant because inflammasome pathways can contribute to bacterial containment in a pathogen-, cell-, and model-dependent manner [25,42,43,87]. A candidate intervention should therefore demonstrate that attenuation of lung injury is retained during antimicrobial treatment without delaying bacterial clearance, increasing extrapulmonary dissemination, or increasing susceptibility to persistent or secondary infection. Supportive measures should likewise be standardized across treatment groups so that improvements in organ function can be attributed to the host-directed intervention rather than differences in resuscitation or supportive care. Therapeutic rather than prophylactic dosing should be prioritized, with the inflammasome-directed intervention initiated after infection is established and evaluated in the context of standard antimicrobial treatment.

Two experimental strategies appear particularly suitable for advancing these concepts. First, ASC2 provides the most direct bat-derived molecular lead [12]. Rather than immediately pursuing systemic ASC inhibition, an initial strategy would be to test transient delivery of bat ASC2 or an ASC2-derived inhibitory construct in an airway-initiated bacterial pneumonia model, followed by validation in polymicrobial sepsis. The key question would be whether ASC-dependent inflammasome assembly and lung injury can be reduced without impairing bacterial clearance. Readouts should therefore include ASC speck formation, caspase-1 activation, IL-1β/IL-18 release, GSDMD cleavage, pulmonary barrier injury and oxygenation together with pulmonary and systemic bacterial burden, dissemination, and survival. Macrophage-directed or lung-restricted delivery would be particularly informative because it could reduce systemic suppression of protective inflammasome functions.

Second, the broader bat-supported principle of partial inflammasome restraint could be tested using temporally controlled NLRP3 inhibition rather than complete genetic ablation. This strategy is not uniquely derived from bats, but bat studies provide biological support for the hypothesis that reduced NLRP3 responsiveness may be compatible with preserved immune competence [11]. A selective NLRP3 inhibitor could therefore be administered therapeutically after establishment of bacterial pneumonia and initiation of antimicrobial treatment, using dose-ranging and delayed-treatment designs to determine whether a functional “taming zone” can be identified. The same paired endpoints described above should be used to determine whether reductions in inflammasome activity and lung injury occur without deterioration in bacterial clearance or survival. Replication in CLP would then test whether the effect extends beyond a single pathogen and infection route.

## 7. Challenges and Future Directions

Bat-inspired inflammasome modulation is an attractive idea, but it should not be oversold. The first obstacle is biological distance. Bats, mice, and humans differ in lifespan, metabolism, pathogen exposure, immune architecture, and tissue repair programs. A mechanism that protects a bat from virus-induced immunopathology may not behave the same way in an elderly patient with bacterial pneumonia, shock, diabetes, and renal failure. This sounds obvious, but it is easy to forget when a pathway looks elegant. Bat NLRP3 dampening and bat ASC2-mediated inflammasome suppression provide powerful concepts, not ready-made sepsis therapies [11,12].

Timing is the second problem. Sepsis is not one immune state. Hyperinflammation and immunosuppression may coexist, but their balance changes over days [9]. This is not just theory. In a 20-year real-world cohort of 1023 septic shock patients, 28-day mortality was 29%, 90-day mortality was 37%, and ICU-acquired infection occurred in 19% [85]. mHLA-DR was markedly depressed during the first ICU week; by day 5/7, the median value was 6603 AB/C and 62% of patients remained below 8000 AB/C. Trajectory analysis identified a “non-improver” cluster comprising 55% of patients, with median mHLA-DR values persistently below 4500 AB/C and higher mortality and ICU-acquired infection rates [85]. These data make one point clear: an anti-inflammasome intervention given without immune monitoring could easily hit the wrong patient at the wrong time. Current sepsis management still relies heavily on antimicrobial therapy, source control, hemodynamic support, and organ support [4]. Any host-directed strategy has to fit into that clinical reality, not replace it.

Patient selection is another unresolved issue. The field has moved toward immune endotyping, but endotypes are not yet routinely used to guide anti-inflammatory treatment at the bedside. Transcriptomic studies have identified sepsis subgroups with distinct immune programs and different outcomes, and clinical phenotyping has suggested that some subgroups may respond differently to therapies [34,35,36]. Still, a usable “inflammasome-high septic lung injury” phenotype has not been firmly established. That is a major gap. It will require integrated biomarkers: circulating IL-1β/IL-18, cleaved gasdermin D, extracellular ASC specks, monocyte HLA-DR, macrophage transcriptional signatures, and perhaps spatial evidence of inflammatory amplification within lung tissue [2,8,77]. No single marker will be enough.

Model choice also matters. LPS-induced lung injury is useful for dissecting inflammatory mechanisms, but it cannot fully reproduce polymicrobial infection, vascular dysfunction, antibiotic exposure, or the temporal complexity of human sepsis. CLP models are closer to abdominal polymicrobial sepsis, yet their relevance to primary septic lung injury is still imperfect. Human macrophage–epithelial–endothelial co-culture systems, lung organoids, precision-cut lung slices, and spatial single-cell profiling of patient samples may help bridge this gap. The next step should not be another isolated pathway diagram. It should be a translational testing pipeline.

Future work should therefore ask three practical questions. Which macrophage states actually drive inflammasome-dependent lung injury? When does their activity become harmful rather than protective? Can bat-inspired regulators—especially ASC-centered approaches—reduce tissue injury while preserving microbial control? These questions are not glamorous, but they are necessary. If they can be answered, bat-inspired inflammasome “taming” may become more than a metaphor. It may become a rational host-directed strategy for septic lung injury.

## 8. Conclusions

Macrophage inflammasomes occupy an awkward but important position in sepsis-associated lung injury. They are not simply pathological machinery waiting to be blocked. They sense danger, help organize early host defense, and shape antimicrobial inflammation. Yet, once their activation becomes excessive or poorly timed, the same system can drive IL-1β/IL-18 release, pyroptosis, ASC speck propagation, neutrophil recruitment, barrier leakage, and ultimately respiratory failure. This is why the language of “taming” matters. It captures the need for restraint without implying total suppression.

Bats offer a rare biological example of this restraint. Their immune systems can tolerate repeated viral threats while limiting inflammasome-driven inflammation, partly through dampened NLRP3 responsiveness and potent ASC2-mediated inhibition of ASC-dependent inflammasomes [11,12]. These mechanisms should not be copied uncritically into human sepsis. The species gap is real. So is the danger of immunosuppression. Still, bat biology gives sepsis research a useful design principle: separate protective host defense from damaging immunopathology whenever possible.

Future therapies for septic lung injury may therefore need to move beyond broad anti-inflammatory strategies. A more precise approach would identify inflammasome-active macrophage states, define the right therapeutic window, and intervene at selected control points—NLRP3 priming, ASC assembly, gasdermin-mediated pyroptosis, or IL-1 family signaling. The goal is not silence. The goal is controlled inflammation. That may be the real lesson from bats.

## Data Availability

No new data were created or analyzed in this study. Data sharing is not applicable to this article.

## References

[1] Singer M., Deutschman C.S., Seymour C.W., Shankar-Hari M., Annane D., Bauer M., Bellomo R., Bernard G.R., Chiche J.D., Coopersmith C.M. (2016). The Third International Consensus Definitions for Sepsis and Septic Shock (Sepsis-3). JAMA.

[2] Matthay M.A., Arabi Y., Arroliga A.C., Bernard G., Bersten A.D., Brochard L.J., Calfee C.S., Combes A., Daniel B.M., Ferguson N.D. (2024). A New Global Definition of Acute Respiratory Distress Syndrome. Am. J. Respir. Crit. Care Med..

[3] Huppert L.A., Matthay M.A., Ware L.B. (2019). Pathogenesis of Acute Respiratory Distress Syndrome. Semin. Respir. Crit. Care Med..

[4] Evans L., Rhodes A., Alhazzani W., Antonelli M., Coopersmith C.M., French C., Machado F.R., McIntyre L., Ostermann M., Prescott H.C. (2021). Surviving sepsis campaign: International guidelines for management of sepsis and septic shock 2021. Intensive Care Med..

[5] Murray P.J., Wynn T.A. (2011). Protective and pathogenic functions of macrophage subsets. Nat. Rev. Immunol..

[6] Broz P., Dixit V.M. (2016). Inflammasomes: Mechanism of assembly, regulation and signalling. Nat. Rev. Immunol..

[7] Mangan M.S.J., Olhava E.J., Roush W.R., Seidel H.M., Glick G.D., Latz E. (2018). Targeting the NLRP3 inflammasome in inflammatory diseases. Nat. Rev. Drug Discov..

[8] McVey M.J., Steinberg B.E., Goldenberg N.M. (2021). Inflammasome activation in acute lung injury. Am. J. Physiol. Lung Cell. Mol. Physiol..

[9] van der Poll T., Shankar-Hari M., Wiersinga W.J. (2021). The immunology of sepsis. Immunity.

[10] Irving A.T., Ahn M., Goh G., Anderson D.E., Wang L.F. (2021). Lessons from the host defences of bats, a unique viral reservoir. Nature.

[11] Ahn M., Anderson D.E., Zhang Q., Tan C.W., Lim B.L., Luko K., Wen M., Chia W.N., Mani S., Wang L.C. (2019). Dampened NLRP3-mediated inflammation in bats and implications for a special viral reservoir host. Nat. Microbiol..

[12] Ahn M., Chen V.C., Rozario P., Ng W.L., Kong P.S., Sia W.R., Kang A.E.Z., Su Q., Nguyen L.H., Zhu F. (2023). Bat ASC2 suppresses inflammasomes and ameliorates inflammatory diseases. Cell.

[13] Dolinay T., Kim Y.S., Howrylak J., Hunninghake G.M., An C.H., Fredenburgh L., Massaro A.F., Rogers A., Gazourian L., Nakahira K. (2012). Inflammasome-regulated cytokines are critical mediators of acute lung injury. Am. J. Respir. Crit. Care Med..

[14] Shi J., Zhao Y., Wang K., Shi X., Wang Y., Huang H., Zhuang Y., Cai T., Wang F., Shao F. (2015). Cleavage of GSDMD by inflammatory caspases determines pyroptotic cell death. Nature.

[15] Kayagaki N., Stowe I.B., Lee B.L., O’Rourke K., Anderson K., Warming S., Cuellar T., Haley B., Roose-Girma M., Phung Q.T. (2015). Caspase-11 cleaves gasdermin D for non-canonical inflammasome signalling. Nature.

[16] Franklin B.S., Bossaller L., De Nardo D., Ratter J.M., Stutz A., Engels G., Brenker C., Nordhoff M., Mirandola S.R., Al-Amoudi A. (2014). The adaptor ASC has extracellular and ‘prionoid’ activities that propagate inflammation. Nat. Immunol..

[17] Wang F., Chen M., Ma J., Wang C., Wang J., Xia H., Zhang D., Yao S. (2022). Integrating bulk and single-cell sequencing reveals the phenotype-associated cell subpopulations in sepsis-induced acute lung injury. Front. Immunol..

[18] Song H., Peng L., Yang Y., Sun K., Chu Y., Deng M., Meng X., Wei H., Cai J., Liu Y. (2026). Dual targeting of NCF1 and NLRP3 by roburic acid orchestrates redox homeostasis and inhibits macrophage death in septic lung injury. Redox Biol..

[19] Zhao X., Xie J., Duan C., Wang L., Si Y., Liu S., Wang Q., Wu D., Wang Y., Yin W. (2024). ADAR1 protects pulmonary macrophages from sepsis-induced pyroptosis and lung injury through miR-21/A20 signaling. Int. J. Biol. Sci..

[20] Park I., Kim M., Choe K., Song E., Seo H., Hwang Y., Ahn J., Lee S.H., Lee J.H., Jo Y.H. (2019). Neutrophils disturb pulmonary microcirculation in sepsis-induced acute lung injury. Eur. Respir. J..

[21] Adams J., Li T., Zhu P., Garbe C., Tu F., Casteel J., Yakubenko V., Williams D.L., Li C., Wang X. (2026). β-Glucan Protects Against Sepsis-Induced Kupffer Cell Loss by Inhibiting Pyroptosis and Promoting Self-Renewal. Immunology.

[22] Ouyang H., Wang Y., Wu J., Ji Y. (2023). Mechanisms of pulmonary microvascular endothelial cells barrier dysfunction induced by LPS: The roles of ceramides and the Txnip/NLRP3 inflammasome. Microvasc. Res..

[23] Yan C., Lin X., Guan J., Ding W., Yue Z., Tang Z., Meng X., Zhao B., Song Z., Li D. (2025). SIRT3 Deficiency Promotes Lung Endothelial Pyroptosis Through Impairing Mitophagy to Activate NLRP3 Inflammasome During Sepsis-Induced Acute Lung Injury. Mol. Cell. Biol..

[24] Tannahill G.M., Curtis A.M., Adamik J., Palsson-McDermott E.M., McGettrick A.F., Goel G., Frezza C., Bernard N.J., Kelly B., Foley N.H. (2013). Succinate is an inflammatory signal that induces IL-1β through HIF-1α. Nature.

[25] Kumari P., Russo A.J., Wright S.S., Muthupalani S., Rathinam V.A. (2021). Hierarchical cell-type-specific functions of caspase-11 in LPS shock and antibacterial host defense. Cell Rep..

[26] Jorgensen I., Miao E.A. (2015). Pyroptotic cell death defends against intracellular pathogens. Immunol. Rev..

[27] Jeznach A., Sidor-Dzitkowska K., Bandyszewska M., Grzanka M., Popławski P., Marszalik A., Domagała-Kulawik J., Stachowiak R., Hoser G., Skirecki T. (2025). Sepsis-induced inflammasome impairment facilitates development of secondary A. baumannii pneumonia. Emerg. Microbes Infect..

[28] Rathkey J.K., Zhao J., Liu Z., Chen Y., Yang J., Kondolf H.C., Benson B.L., Chirieleison S.M., Huang A.Y., Dubyak G.R. (2018). Chemical disruption of the pyroptotic pore-forming protein gasdermin D inhibits inflammatory cell death and sepsis. Sci. Immunol..

[29] Jia A., Wang Y., Zhou R., Han L. (2025). USP48 promotes NLRP3-dependent pyroptosis of alveolar macrophages to exacerbate sepsis-induced acute lung injury. Int. Immunopharmacol..

[30] Zhu C., Zhang W., Liao Y., Jiang R., Cheng L., Wang Y., Zhou T., Tao Y., Xu S., Yu Y. (2026). ERK/Smurf1 regulates non-canonical pyroptosis by prompting Caspase-11 ubiquitination. Cell Death Differ..

[31] Liu W., Zhang T., Wang B., Yin H. (2026). Aerobic glycolysis promotes NLRP3 inflammasome activation via NLRP3 lactylation. Cell Chem. Biol..

[32] Wiersinga W.J., van der Poll T. (2022). Immunopathophysiology of human sepsis. eBioMedicine.

[33] Hotchkiss R.S., Monneret G., Payen D. (2013). Sepsis-induced immunosuppression: From cellular dysfunctions to immunotherapy. Nat. Rev. Immunol..

[34] Seymour C.W., Kennedy J.N., Wang S., Chang C.H., Elliott C.F., Xu Z., Berry S., Clermont G., Cooper G., Gomez H. (2019). Derivation, Validation, and Potential Treatment Implications of Novel Clinical Phenotypes for Sepsis. JAMA.

[35] Scicluna B.P., van Vught L.A., Zwinderman A.H., Wiewel M.A., Davenport E.E., Burnham K.L., Nürnberg P., Schultz M.J., Horn J., Cremer O.L. (2017). Classification of patients with sepsis according to blood genomic endotype: A prospective cohort study. Lancet Respir. Med..

[36] Sweeney T.E., Azad T.D., Donato M., Haynes W.A., Perumal T.M., Henao R., Bermejo-Martin J.F., Almansa R., Tamayo E., Howrylak J.A. (2018). Unsupervised Analysis of Transcriptomics in Bacterial Sepsis Across Multiple Datasets Reveals Three Robust Clusters. Crit. Care Med..

[37] Shankar-Hari M., Calandra T., Soares M.P., Bauer M., Wiersinga W.J., Prescott H.C., Knight J.C., Baillie K.J., Bos L.D.J., Derde L.P.G. (2024). Reframing sepsis immunobiology for translation: Towards informative subtyping and targeted immunomodulatory therapies. Lancet Respir. Med..

[38] Shakoory B., Carcillo J.A., Chatham W.W., Amdur R.L., Zhao H., Dinarello C.A., Cron R.Q., Opal S.M. (2016). Interleukin-1 Receptor Blockade Is Associated with Reduced Mortality in Sepsis Patients with Features of Macrophage Activation Syndrome: Reanalysis of a Prior Phase III Trial. Crit. Care Med..

[39] Medzhitov R., Schneider D.S., Soares M.P. (2012). Disease tolerance as a defense strategy. Science.

[40] Martins R., Carlos A.R., Braza F., Thompson J.A., Bastos-Amador P., Ramos S., Soares M.P. (2019). Disease Tolerance as an Inherent Component of Immunity. Annu. Rev. Immunol..

[41] Hu J.J., Liu X., Xia S., Zhang Z., Zhang Y., Zhao J., Ruan J., Luo X., Lou X., Bai Y. (2020). FDA-approved disulfiram inhibits pyroptosis by blocking gasdermin D pore formation. Nat. Immunol..

[42] Perlee D., de Beer R., Florquin S., van der Poll T., Veer C.V., de Vos A.F. (2020). Caspase-11 contributes to pulmonary host defense against *Klebsiella pneumoniae* and local activation of coagulation. Am. J. Physiol. Lung Cell. Mol. Physiol..

[43] Zhou Y., Chai Z., Pandeya A., Yang L., Zhang Y., Zhang G., Wu C., Li Z., Wei Y. (2025). Caspase-11 and NLRP3 exacerbate systemic Klebsiella infection through reducing mitochondrial ROS production. Front. Immunol..

[44] Calisher C.H., Childs J.E., Field H.E., Holmes K.V., Schountz T. (2006). Bats: Important reservoir hosts of emerging viruses. Clin. Microbiol. Rev..

[45] Li W., Shi Z., Yu M., Ren W., Smith C., Epstein J.H., Wang H., Crameri G., Hu Z., Zhang H. (2005). Bats are natural reservoirs of SARS-like coronaviruses. Science.

[46] Leroy E.M., Kumulungui B., Pourrut X., Rouquet P., Hassanin A., Yaba P., Délicat A., Paweska J.T., Gonzalez J.P., Swanepoel R. (2005). Fruit bats as reservoirs of Ebola virus. Nature.

[47] Letko M., Seifert S.N., Olival K.J., Plowright R.K., Munster V.J. (2020). Bat-borne virus diversity, spillover and emergence. Nat. Rev. Microbiol..

[48] Olival K.J., Hosseini P.R., Zambrana-Torrelio C., Ross N., Bogich T.L., Daszak P. (2017). Erratum: Host and viral traits predict zoonotic spillover from mammals. Nature.

[49] Morales A.E., Dong Y., Brown T., Baid K., Kontopoulos D., Gonzalez V., Huang Z., Ahmed A.W., Bhuinya A., Hilgers L. (2025). Bat genomes illuminate adaptations to viral tolerance and disease resistance. Nature.

[50] Zhang G., Cowled C., Shi Z., Huang Z., Bishop-Lilly K.A., Fang X., Wynne J.W., Xiong Z., Baker M.L., Zhao W. (2013). Comparative analysis of bat genomes provides insight into the evolution of flight and immunity. Science.

[51] Jebb D., Huang Z., Pippel M., Hughes G.M., Lavrichenko K., Devanna P., Winkler S., Jermiin L.S., Skirmuntt E.C., Katzourakis A. (2020). Six reference-quality genomes reveal evolution of bat adaptations. Nature.

[52] Zhou P., Tachedjian M., Wynne J.W., Boyd V., Cui J., Smith I., Cowled C., Ng J.H., Mok L., Michalski W.P. (2016). Contraction of the type I IFN locus and unusual constitutive expression of IFN-α in bats. Proc. Natl. Acad. Sci. USA.

[53] Pavlovich S.S., Lovett S.P., Koroleva G., Guito J.C., Arnold C.E., Nagle E.R., Kulcsar K., Lee A., Thibaud-Nissen F., Hume A.J. (2018). The Egyptian Rousette Genome Reveals Unexpected Features of Bat Antiviral Immunity. Cell.

[54] Ahn M., Cui J., Irving A.T., Wang L.F. (2016). Unique Loss of the PYHIN Gene Family in Bats Amongst Mammals: Implications for Inflammasome Sensing. Sci. Rep..

[55] Xie J., Li Y., Shen X., Goh G., Zhu Y., Cui J., Wang L.F., Shi Z.L., Zhou P. (2018). Dampened STING-Dependent Interferon Activation in Bats. Cell Host Microbe.

[56] Fernandes-Alnemri T., Yu J.W., Juliana C., Solorzano L., Kang S., Wu J., Datta P., McCormick M., Huang L., McDermott E. (2010). The AIM2 inflammasome is critical for innate immunity to Francisella tularensis. Nat. Immunol..

[57] Rathinam V.A., Jiang Z., Waggoner S.N., Sharma S., Cole L.E., Waggoner L., Vanaja S.K., Monks B.G., Ganesan S., Latz E. (2010). The AIM2 inflammasome is essential for host defense against cytosolic bacteria and DNA viruses. Nat. Immunol..

[58] Gorbunova V., Seluanov A., Kennedy B.K. (2020). The World Goes Bats: Living Longer and Tolerating Viruses. Cell Metab..

[59] Goh G., Ahn M., Zhu F., Lee L.B., Luo D., Irving A.T., Wang L.F. (2020). Complementary regulation of caspase-1 and IL-1β reveals additional mechanisms of dampened inflammation in bats. Proc. Natl. Acad. Sci. USA.

[60] Lu A., Magupalli V.G., Ruan J., Yin Q., Atianand M.K., Vos M.R., Schröder G.F., Fitzgerald K.A., Wu H., Egelman E.H. (2014). Unified polymerization mechanism for the assembly of ASC-dependent inflammasomes. Cell.

[61] Minton K. (2023). Negative regulation of inflammasome activation in bats. Nat. Rev. Immunol..

[62] Kessler S., Burke B., Andrieux G., Schinköthe J., Hamberger L., Kacza J., Zhan S., Reasoner C., Dutt T.S., Kaukab Osman M. (2024). Deciphering bat influenza H18N11 infection dynamics in male Jamaican fruit bats on a single-cell level. Nat. Commun..

[63] Mandl J.N., Schneider C., Schneider D.S., Baker M.L. (2018). Going to Bat(s) for Studies of Disease Tolerance. Front. Immunol..

[64] Huang Z., Whelan C.V., Foley N.M., Jebb D., Touzalin F., Petit E.J., Puechmaille S.J., Teeling E.C. (2019). Longitudinal comparative transcriptomics reveals unique mechanisms underlying extended healthspan in bats. Nat. Ecol. Evol..

[65] Gorbunova V., Seluanov A., Zhang Z., Gladyshev V.N., Vijg J. (2014). Comparative genetics of longevity and cancer: Insights from long-lived rodents. Nat. Rev. Genet..

[66] Toshkova N., Zhelyzkova V., Reyes-Ruiz A., Haerens E., de Castro Deus M., Lacombe R.V., Lecerf M., Gonzalez G., Jouvenet N., Planchais C. (2024). Temperature sensitivity of bat antibodies links metabolic state of bats with antigen-recognition diversity. Nat. Commun..

[67] Hagar J.A., Powell D.A., Aachoui Y., Ernst R.K., Miao E.A. (2013). Cytoplasmic LPS activates caspase-11: Implications in TLR4-independent endotoxic shock. Science.

[68] Downs K.P., Nguyen H., Dorfleutner A., Stehlik C. (2020). An overview of the non-canonical inflammasome. Mol. Asp. Med..

[69] Matikainen S., Nyman T.A., Cypryk W. (2020). Function and Regulation of Noncanonical Caspase-4/5/11 Inflammasome. J. Immunol..

[70] Coll R.C., Robertson A.A., Chae J.J., Higgins S.C., Muñoz-Planillo R., Inserra M.C., Vetter I., Dungan L.S., Monks B.G., Stutz A. (2015). A small-molecule inhibitor of the NLRP3 inflammasome for the treatment of inflammatory diseases. Nat. Med..

[71] Corcoran S.E., Halai R., Cooper M.A. (2021). Pharmacological Inhibition of the Nod-Like Receptor Family Pyrin Domain Containing 3 Inflammasome with MCC950. Pharmacol. Rev..

[72] Klück V., Jansen T., Janssen M., Comarniceanu A., Efdé M., Tengesdal I.W., Schraa K., Cleophas M.C.P., Scribner C.L., Skouras D.B. (2020). Dapansutrile, an oral selective NLRP3 inflammasome inhibitor, for treatment of gout flares: An open-label, dose-adaptive, proof-of-concept, phase 2a trial. Lancet Rheumatol..

[73] Giamarellos-Bourboulis E.J., Kotsaki A., Kotsamidi I., Efthymiou A., Koutsoukou V., Ehler J., Paridou A., Frantzeskaki F., Müller M.C.A., Pickkers P. (2026). Precision Immunotherapy to Improve Sepsis Outcomes: The ImmunoSep Randomized Clinical Trial. JAMA.

[74] Hsu C.G., Chávez C.L., Zhang C., Sowden M., Yan C., Berk B.C. (2022). The lipid peroxidation product 4-hydroxynonenal inhibits NLRP3 inflammasome activation and macrophage pyroptosis. Cell Death Differ..

[75] Yang R., Zheng T., Xiang H., Liu M., Hu K. (2024). Lung single-cell RNA profiling reveals response of pulmonary capillary to sepsis-induced acute lung injury. Front. Immunol..

[76] Mould K.J., Barthel L., Mohning M.P., Thomas S.M., McCubbrey A.L., Danhorn T., Leach S.M., Fingerlin T.E., O’Connor B.P., Reisz J.A. (2017). Cell Origin Dictates Programming of Resident versus Recruited Macrophages during Acute Lung Injury. Am. J. Respir. Cell Mol. Biol..

[77] Mould K.J., Moore C.M., McManus S.A., McCubbrey A.L., McClendon J.D., Griesmer C.L., Henson P.M., Janssen W.J. (2021). Airspace Macrophages and Monocytes Exist in Transcriptionally Distinct Subsets in Healthy Adults. Am. J. Respir. Crit. Care Med..

[78] Shan M., Zhang S., Luo Z., Deng S., Ran L., Zhou Q., Wan H., Ye J., Qian C., Fan X. (2025). Itaconate promotes inflammatory responses in tissue-resident alveolar macrophages and exacerbates acute lung injury. Cell Metab..

[79] Jiang T., Liu E., Li Z., Yan C., Zhang X., Guan J., Zhan Y., Zhao B., Ding W. (2024). SIRT1-Rab7 axis attenuates NLRP3 and STING activation through late endosomal-dependent mitophagy during sepsis-induced acute lung injury. Int. J. Surg..

[80] Xie F., Fan J.X., Wang H., Song Q.L., Xu Y.S., Lan Q.S., Jiang X.M., Cheng J., Hou Y.M., Yang H.R. (2025). Macrophage TRIM21 knockout inhibits septic acute lung injury by downregulating autophagy regulator protein ubiquitination. Autophagy.

[81] Fan Y., Mei J., Shen Y., Gao Y., Zhao L., Meng S., Zhou S., Qian Y., Zhang Y., Wang Z. (2025). Promotion of NLRP3 autophagosome degradation by PV-K nanodevice for protection against macrophage pyroptosis-mediated lung injury. J. Nanobiotechnol..

[82] Wittmann N., Behrendt A.K., Mishra N., Bossaller L., Meyer-Bahlburg A. (2021). Instructions for Flow Cytometric Detection of ASC Specks as a Readout of Inflammasome Activation in Human Blood. Cells.

[83] Cui J., Oehrl S., Ahmad F., Brenner T., Uhle F., Nusshag C., Rupp C., Funck F., Meisel S., Weigand M.A. (2020). Detection of In Vivo Inflammasome Activation for Predicting Sepsis Mortality. Front. Immunol..

[84] Basiorka A.A., McGraw K.L., Abbas-Aghababazadeh F., McLemore A.F., Vincelette N.D., Ward G.A., Eksioglu E.A., Sallman D.A., Ali N.A., Padron E. (2018). Assessment of ASC specks as a putative biomarker of pyroptosis in myelodysplastic syndromes: An observational cohort study. Lancet Haematol..

[85] Monneret G., Lafon T., Gossez M., Evrard B., Bodinier M., Rimmelé T., Argaud L., Cour M., Friggeri A., Lepape A. (2025). Publisher Correction: Monocyte HLA-DR expression in septic shock patients: Insights from a 20-year real-world cohort of 1023 cases. Intensive Care Med..

[86] Rittirsch D., Huber-Lang M.S., Flierl M.A., Ward P.A. (2009). Immunodesign of experimental sepsis by cecal ligation and puncture. Nat. Protoc..

[87] Aachoui Y., Leaf I.A., Hagar J.A., Fontana M.F., Campos C.G., Zak D.E., Tan M.H., Cotter P.A., Vance R.E., Aderem A. (2013). Caspase-11 protects against bacteria that escape the vacuole. Science.

